# Meta-analysis of the mutational status of circulation tumor cells and paired primary tumor tissues from colorectal cancer patients

**DOI:** 10.18632/oncotarget.18272

**Published:** 2017-05-26

**Authors:** Yong Liu, Stefano Meucci, Liming Sheng, Ulrich Keilholz

**Affiliations:** ^1^ Surgical Department of Colorectal Cancer, Zhejiang Cancer Hospital, Hangzhou, Zhejiang Province, China; ^2^ Charité Comprehensive Cancer Center, Labor AG Keilholz, Berlin, Germany; ^3^ Department of Radiotherapy, Zhejiang Cancer Hospital, Hangzhou, Zhejiang Province, China

**Keywords:** colorectal cancer, KRAS mutation, BRAF mutation, circulating tumor cells, clinical stage

## Abstract

As predictive markers for anti-EGFR therapy, KRAS and BRAF mutations are routinely detected in primary and metastatic colorectal cancer (CRC) cells, but seldom in circulating tumor cells (CTCs). Detecting mutations in CTCs could help explain mutational differences between tumor cells at local sites and distant metastases, thereby improving treatment outcomes. Here, we conducted a systematic review and meta-analysis to compare KRAS and BRAF mutations in paired CTCs and primary tumors from CRC patients, to detect any possible discordance. A total of 244 CRC patients from nine studies were included. Our subgroup meta-analysis demonstrated that the total odds ratio for mutations in CTCs was only 55% of that in primary tumors in the stage IV subgroup. We also found low heterogeneity among studies and differences in mutations between CTCs and primary tumors in the stage IV subgroup (I^2^ = 0%, *P* = 0.01). We observed a higher frequency of KRAS mutations in CTCs than in primary tumors at early stages (I + II), a similar frequency in stage III, and a lower frequency in stage IV. There were also differences among the Epcam-targeted CTC enrichment, PCR-based mutation profiling, and ≥ 3 CTCs enriched (I^2^ = 0%, *P* = 0.03) subgroups. These finding indicate mutational discordance between CTCs and primary CRCs, particularly in the stage IV and KRAS subgroups. We suggest large-sample studies stratified by clinical stage and KRAS subtype are urgently warranted to accurately evaluate mutational variations in CTCs compared to primary and metastatic CRC cells.

## INTRODUCTION

Colorectal cancer (CRC) is the third leading cause of cancer-related death worldwide [1]. The endothelial growth factor receptor (EGFR) relative pathway regulates the expression of genes involved in the proliferation, angiogenesis, and metastasis of CRC cells [2, 3]. Mutations in the Kirsten rat sarcoma (KRAS) viral oncogene have been reported to be present in 30–40% of CRC patients and to correlate with clinical resistance to anti-EGFR drugs in metastatic CRCs [4–6]. Indeed, KRAS mutations are a predictive biomarker for anti-EGFR resistance in metastatic CRC (mCRC) [7, 8]. Furthermore, KRAS mutations in primary tumors correlate with poor prognosis and short term survival [9, 10]. Therefore, only CRC patients with wild type KRAS usually receive anti-EGFR therapy [11, 12]. BRAF (proto-oncogeneB-Raf), also part of the EGFR pathway, is also mutated in 10% of mCRC patients [8, 13, 14]. In addition, retrospective analyses have shown that wild-type BRAF is necessary for successful response to anti-EGFR therapies in metastatic CRCs (mCRCs) [13, 15]. KRAS and BRAF mutations together serve as predictive markers for anti-EGFR therapies [16, 17].

Recently, studies exploring the correlation between KRAS mutational status in primary and metastatic tumors of CRC patients revealed intra-tumor heterogeneity or mutation discordance after anti-EGFR treatment failure in patients with wild type KRAS [18–21]. Furthermore, other studies suggested that resistance to anti-EGFR therapy possibly stemmed from selection of preexisting minor sub-clones harboring mutations [5, 11]. The mutational status of metastatic tumors does not always correspond with those in primary lesions. Circulating tumor cells (CTCs) can carry mutant variants from local tumor sites to distant metastases [22]. Recent improvements in DNA-sequencing technology have allowed high resolution by exploring sub-clone heterogeneity between primary and metastatic tumors [23, 24], but recovering sufficient tumor cells is still challenging to exceed the available sequencing analytical platforms [25]. Genotyping of CTCs might improve the monitoring of response to targeted EGFR therapies by identifying genomic profiles and predicting disease metastasis prior to clinical progression [26–29]. High concordance in mutations has been observed between CTCs and primary tissues in mCRC patients and the presumption that discordance exists in both wild type and mutation sub-populations has been challenged [30–34]. It is also possible that mutated sub-clones in primary tumors shed as CTCs into peripheral blood were not present in the tumor tissues used for genomic analyses [35–38]. On the other hand, some studies have detected mutations in CTCs at the sub-clone level by deep sequencing of primary tumor tissues [29]. However, based on genotype and phenotype profiling, accurate detection of the mutational status of CTCs is still challenging [39]. Here, we tested for KRAS and BRAF mutations in CTCs and paired primary CRCs. We also investigated genetic heterogeneity between CTCs and primary tumors.

## MATERIALS AND METHODS

The protocol of our system review was published in PROSPERO: CRD42016042107

### Search strategy

The identification of potentially relevant studies was performed through a comprehensive and systematic search in PubMed, EMBASE, Web of Science Databases, and Google Scholar by using the following keywords “colorectal cancer”, “circulating tumor cells” and “mutation”. Searching details: “neoplastic cells, circulating” [MeSH Terms] OR (“neoplastic” [All Fields] AND “cells” [All Fields] AND “circulating” [All Fields]) OR “circulating neoplastic cells” [All Fields] OR (“circulating” [All Fields] AND “tumor” [All Fields] AND “cell” [All Fields]) OR “circulating tumor cell” [All Fields] AND (“colorectal neoplasms” [MeSH Terms] OR (“colorectal” [All Fields] AND “neoplasms” [All Fields]) OR “colorectal neoplasms” [All Fields] OR (“colorectal” [All Fields] AND “cancer” [All Fields]) OR “colorectal cancer” [All Fields]) AND “mutation” [MeSH Terms] OR “mutation” [All Fields]. The latest search was updated on September 30, 2016. Bibliographies of eligible studies, review articles, the reference lists of each selected study, and other relevant publications were also reviewed to identify all potentially relevant studies.

### Inclusion and exclusion criteria

To qualify as relevant, a study had to fulfill the following criteria: (1) circulating tumor cells were enriched and isolated from CRC patients; (2) KRAS and BRAF mutations had to be present in CTCs and primary tumors; (3) the correlations of KRAS mutation in CTCs and paired primary tissues were assessed; (4) the correlations of KRAS mutation in CTCs and paired primary tissues based on treatment outcome (progression or stable) were assessed; (5) the correlations of BRAF mutation in CTCs and paired primary tissues were assessed; (6) to have been published as a full paper in English up until September 30, 2016. Studies were excluded from our analysis if any of the following conditions occurred: (1) not analyzing KRAS or BRAF mutation in CTCs; (2) CTCs-related analyses without KRAS or BRAF mutation; (3) the samples for KRAS or BRAF mutation analyses were not from CRC patients; (4) small sample analysis (less than four cases); (5) non-human sample analysis.

### Data extraction and quality assessment

Two investigators independently screened the studies and extracted the data from included studies by using standard data abstraction forms. For each study, relevant data were compiled as follows: name of first author, year of publication, total number of patients included, patients’ gender, tumor location (colon/rectum), clinical stage, time of blood sample draws, number of patients with CTCs detected, cutoff number of CTCs, enrichment method and antibody staining of CTCs, number of mutations in CTCs, tissue samples, and in both CTCs and tissue samples combined, subtype mutation of CTCs, methods for mutation detection, tumor status (stable vs progression), and NOS score. The mutational status of paired CTCs and tumor tissue samples from each patient along with clinical data were evaluated. Then the data from a total of nine qualifying studies were used for further analysis. The quality of each study was independently assessed by two investigators using the Newcastle–Ottawa Scale (NOS) [40].

### Statistical methods

Data were presented as odds ratio (OR) with its 95% confidence interval(CI) to show the agreement of KRAS and BRAF mutation in paired CTCs and primary tumor samples while risk ratios (RR) at 95% CI were presented to show the agreement of KRAS mutation in paired CTCs and tissue samples based on tumors status (stable or progression). The individual OR and RR were combined into pooled ORs and RRs, and initial analyses were performed with a fixed effect model assuming homogeneity in the individual studies. Heterogeneity was evaluated by *Q*-test and I^2^ test. When statistically significant heterogeneity among the studies was found by *Q*-test (*P <* 0.05) or I^2^ > 50%, subgroups were classified by gene subtype, stage, and CTC enrichment, with isolation approach stratification or random effect models being applied for further meta-analysis. Otherwise, the fixed effects model was adopted. Sensitivity analyses were conducted to identify whether results of the meta-analysis were affected by exclusion of any individual study and to testify the reliability of the conclusions. All *P* values were 2-sided and all analyses were performed using Review Manager 5.3.

## RESULTS

### Overview of included studies and quality assessment

**Figure 1:**
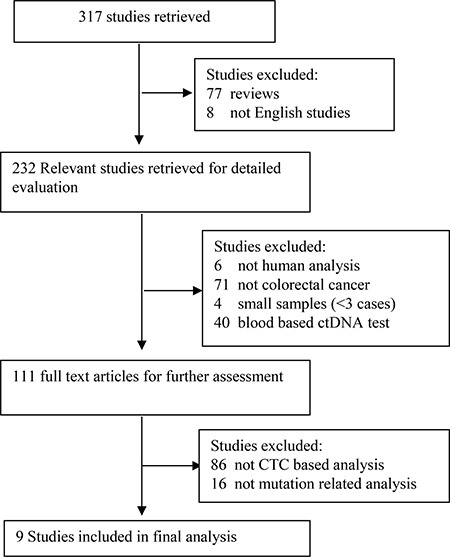
Diagram for retrieval of studies

From 317 studies retrieved, nine studies that focus on comparing CTC-related mutations with paired tumor tissue of CRCs were included for systematic review (See Figure 1, Table 1). Table 1 summarizes details as name of first author (year of publication), total number of patients included, gender, tumor location, clinical stage, time of blood sample draws, number of patients with CTCs, cutoff number of CTCs, enrichment method and antibody staining of CTCs, number of mutation in CTCs, tissue samples, and in both CTCs and tissue samples combined, subtype mutation of CTCs, methods for mutation detection, tumor status, and NOS score. According to the NOS quality assessment, all the selected studies have high quality with a median score of 8.11 stars and were thus subject to further meta-analyses. Among the total 315 CRC patients, 181 (57.46%) were males and 134 (42.54%) females, with 228 (72.38%) colon carcinomas and 87 (27.62%) rectal carcinomas. Among 315 CRC patients, KRAS and BRAF mutations were detected in CTCs and primary tumors from 244 CRC patients. KRAS codon12 and codon13 mutations were detected in 28.27% (69/244) and 5.73% (14/244) of the cases, separately, while BRAF mutations were detected in 5.11% (7/137) of the cases. According to the UICC Classification of Colorectal Cancer, eight cases (3.28%) were classified as stage I, 24 cases (9.83%) as stage II, 47 cases (19.26 %) as stage III, and stage IV had the majority population with 165 cases (67.62%). The concordance of KRAS mutation in CTCs with paired primary tissues of CRCs was compared in all nine studies, but only four studies were evaluated for BRAF mutation of CTCs, three studies were analyzed for KRAS mutation of CTCs with tumor status, and one study assessed mutations among primary tumors, CTCs, and metastatic lesions [30–38].

**Table 1:** Characteristics of studies involved in mutation analysis of CTCs

First author (year of publication)	1 Buim, M.E (2015)	2 Fabbri, F (2013)	3 Harb, W (2013)	4 Raimondi, C (2014)	5 Kalikaki, A (2014)	6 Lyberopoulou, A (2015)	7 Mostert, B (2013)	8 Steinert G (2014)	9 Mohamed Suhaimi, N.A (2015)	Total
Total number of patients included	26	40	15	40	31	52	43	28	40	315
Patients’ gender (male/female)	18/8	24/16	11/4	15/25	22/9	33/19	28/15	11/17	19/21	181/134
Tumor location (colon/rectal)	16 /10	29/11	12/3	28/12	31/0	41/11	26/17	23/5	22/18	228/87
Number of patients with CTCs detected	21	16	14	30	23	52	26	22	40	244
Clinical stage	IV 21	IV 16	II 4 III 2 IV 8	IV 30	IV 23	II 6 III 24 IV 22	IV 26	III 6 IV 16	I 8 II 14 III 15 IV 3	I 8 II 24 III 47 IV 165
Time of blood sample draws	After chemotherapy and monoclonal therapy	Before treatment	After operation	After chemotherapy and monoclonal therapy	After chemotherapy and monoclonal therapy	Before treatment	Before tumor resection	Before or in the operation	After operation	
Cutoff number of CTCs	2 cells	5 cell	5 cells	1 cell	1 cell	2 cells	3 cells	2 cells	1 cell	
Enrichment and antibody staining of CTCs	Isolation by size of epithelial tumors ( ISET) CD45- cells	Density gradient centrifugation CK +/Hoechst +/ CD45- cells	IsoFlux System EpCAM+ cell	CellSearch EPCAM+/CK+/ DAPI +/CD45- cells	CellSearch EPCAM+/CK+/ DAPI +/CD45- cells	Density gradient centrifugation EpCAM+/ Vimentin+/CK+/CD45- cells	CellSearch TM EPCAM+	CellSearch System EPCAM+/CK+ cells	Size-based filtration unit CK+/DAPI+/ CD45- cell	
Number of mutation in CTCs detected	KRAS: codons12 7/21	KRAS: codons12 3/16	KRAS: codons12 1/14 codons13 5/14	KRAS: codons12 6/30	KRAS: codons12 6/23 codons13 1/23	KRAS: codons12 29/52 codons13 0/52 BRAF: 4/52	KRAS: codons12 4/26 codons13 1/26 BRAF: 1/26	KRAS: codons12 4/22 codons13 2/22 BRAF: 1/19	KRAS: codons12 9/40 codons13 5/40 BRAF: 1/40	KRAS: codons12 69/244 codons13 14/244 BRAF: 7/137
Number of mutation in tissue samples	KRAS: codons12 6/21 codons13 3/21	KRAS: codons12 6/16 codons13 3/16	KRAS: codons12 1/14 codons13 1/14	KRAS: codons12 13/30	KRAS: codons12 7/23 codons13 1/23	KRAS: codons12 26/52 codons13 0/52 BRAF: 6/52	KRAS: codons12 8/26 codons13 1/26 BRAF: 1/26	KRAS: codons12 8/22 codons13 1/22 BRAF: 3/19	KRAS: codons12 9/40 codons13 2/40 BRAF: 5/ 40	KRAS: codons12 84/244 codons13 12/244 BRAF: 15/137
Number of mutations in both CTC and tissue samples	KRAS: 5 cases	KRAS: 2 cases	KRAS: 1 case	KRAS: 2 cases	KRAS: 5 cases	KRAS: 26 cases BRAF: 4 cases	KRAS: 4 cases BRAF: 1 case	KRAS: 5 cases BRAF: 1 case	KRAS: 9 cases BRAF: 1 case	KRAS: 59 cases BRAF: 7 case
Subtype mutation of CTCs	KRAS codons12 codons13	KRAS codons12 codons13	KRAS codons12 codons13	KRAS codons12 codons13	KRAS codons12 codons13	KRAS codons12 codons13 BRAF (V600E)	KRAS codons12 codons13 BRAF (V600E)	KRAS codons12 codons13 BRAF (V600E)	KRAS codons12 codons13 BRAF (V600E)	
Methods for mutative detection of CTCs	Pyrosequencing	Pyrosequencing	castPCR	RT-PCR	PNA-mediated PCR	RFLP assay and ASPCR	nested ASB PCR	aCGH PCR	HRM assay and ASPCR	
Tumor status (progression/stable)	5/16	NA	7/6	NA	6/17	NA	NA	NA	NA	18/39
NOS score	8	8	8	8	9	8	8	8	8	8.11 (mean)

NA: not available; castPCR: Competitive Allele-Specific TaqMan PCR; PNA: peptide nucleic acid; RFLP: restriction fragment length polymorphism; nested ASB PCR: nested Allele-Specific Blocker PCR; aCGH PCR: array comparative genomic hybridization PCR; HRM: High resolution melt; ASPCR: Allele-specific PCR; NOS score: Newcastle–Ottawa Scale score.

### Correlation of KRAS mutation in paired CTCs and primary tumors

We summarized the data from all nine studies based on KRAS mutation with stage and codon subgroup stratification (See Table 2). From the results calculated by McNemar Test and Kappa value, significant discordance (McNemar value < 0.001 for codon12+13 and codon12, 0.289 for codon13) and poor agreement (Kappa value 0.377 for codon12+13, 0.397 for codon12 and 0.476 for codon13) of mutations in paired CTCs and primary CRC tumors were observed in the stage IV subgroup. Conversely, high concordance and better agreement between those two study populations (McNemar value 0.687 for codon12+13 and 0.500 for codon12, Kappa value 0.744 for codon12+13 and 0.913 for codon12) were observed in the stage III subgroup.

Table 2Relationship of KRAS mutation in paired CTCs with tissue samples of CRC patients based on stage III and IV stratification

**Table:** 

	Stage IV							Stage III						
		CTCs (*n*)			McNemar-Test	Kappa	*P* value		CTCs (*n*)			McNemar-Test	Kappa	*P* value
		Mutation Codon 12+13	Wild type	Total					Mutation Codon 12+13	Wild type	Total			
Tissue sample (*n*)	Mutation Codon 12+13	35	39	74	< 0.001*	0.377	< 0.001*	Mutation Codon 12+13	19	2	21	0.687	0.744	< 0.001*
	Wild type	10	81	91				Wild type	4	22	26			
	Total	45	120	165				Total	23	24	47			
		Mutation Codon12	Wild type	Total	McNemar-Test	Kappa	*P* value		Mutation Codon12	Wild type	Total	McNemar-Test	Kappa	*P* value
Tissue sample (*n*)	Mutation Codon12	30	34	64	< 0.001*	0.397	< 0.001*	Mutation Codon12	19	2	21	0.500	0.913	< 0.001*
	Wild type	10	91	101				Wild type	0	26	26			
	Total	40	125	165				Total	19	28	47			
		Mutation Codon13	Wild type	Total	McNemar-Test	Kappa	*P* value		Mutation Codon13	Wild type	Total	McNemar-Test	Kappa	*P* value
Tissue sample (*n*)	Mutation Codon13	4	6	10	0.289	0.476	< 0.001*	Mutation Codon13	0	0	0	NA	NA	NA
	Wild type	2	153	155				Wild type	4	43	47			
	Total	6	159	165				Total	4	43	47			

*:indicate *P* <0.05, NA: not available.

**Table:** 

	Stage IV							Stage III						
		CTCs (*n*)			McNemar-Test	Kappa	*P* value		CTCs (*n*)			McNemar-Test	Kappa	*P* value
		Mutation Codon 12+13	Wild type	Total					Mutation Codon 12+13	Wild type	Total			
Tissue sample (*n*)	Mutation Codon 12+13	33	41	74	< 0.001*	0.321	< 0.001*	Mutation Codon 12+13	17	4	21	1.000	0.614	< 0.001*
	Wild type	13	77	90				Wild type	5	21	26			
	Total	46	118	164				Total	22	25	47			
		Mutation Codon12	Wild type	Total	McNemar-Test	Kappa	*P* value		Mutation Codon12	Wild type	Total	McNemar-Test	Kappa	*P* value
Tissue sample (*n*)	Mutation Codon12	29	36	65	0.001*	0.335	< 0.001*	Mutation Codon12	20	2	22	0.500	0.914	< 0.001*
	Wild type	13	86	99				Wild type	0	25	25			
	Total	42	122	164				Total	20	27	47			
		Mutation Codon13	Wild type	Total	McNemar-Test	Kappa	*P* value		Mutation Codon13	Wild type	Total	McNemar-Test	Kappa	*P* value
Tissue sample (*n*)	Mutation Codon13	5	27	32	< 0.001*	0.147	0.025*	Mutation Codon13	4	0	4	0.125	0.624	< 0.001*
	Wild type	6	126	132				Wild type	4	39	43			
	Total	11	153	164				Total	8	39	47			

### Pooled data analysis of KRAS mutation in paired CTCs and primary tumors (stage I–IV)

**Figure 2:**
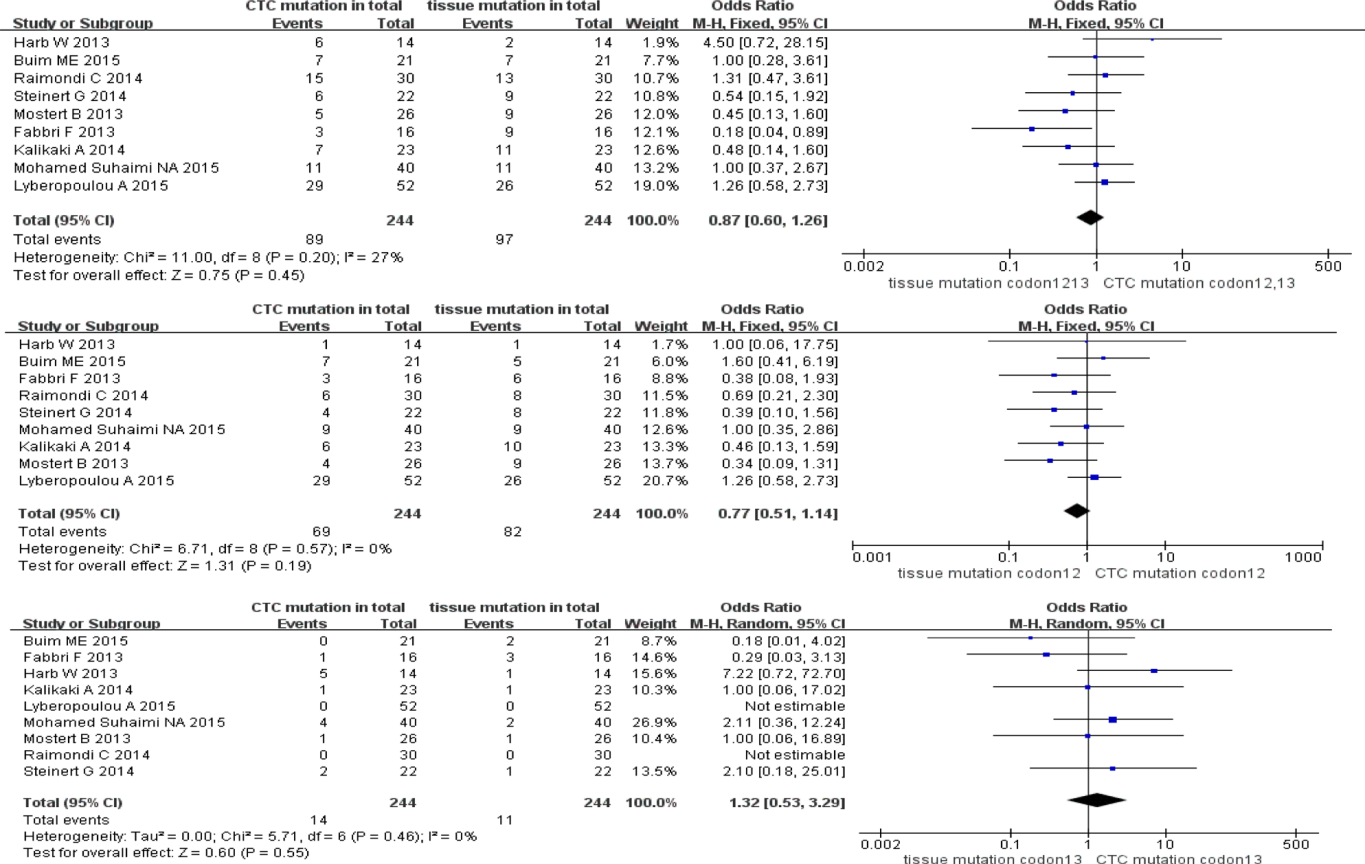
Analyses of KRAS codon12+13, codon12 and codon13 mutation in paired CTCs and primary tumors (stage I-IV)

With reference to stage I-IV CRC patients, the odds ratio (OR) of paired CTCs and tumor tissues was 0.87 (95% CI; 0.60, 1.26) for KRAS codon12+13 mutation, 0.77 (95% CI; 0.51, 1.14) for KRAS codon12 mutation, and 1.32 (95% CI; 0.53, 3.29) for KRAS codon13 mutation (see Figure 2). No heterogeneity or discordance between studies was observed for three KRAS sub-type mutations in CRC patients (I^2^ = 27%, test for overall effect *P* = 0.45 for codon12+13, I^2^ = 0%, *P* = 0.19 for codon12 and I^2^ = 0%, *P* = 0.55 for codon13).

### Pooled data analysis of KRAS sub-type mutation in paired CTCs and primary tumors (stage IV)

**Figure 3:**
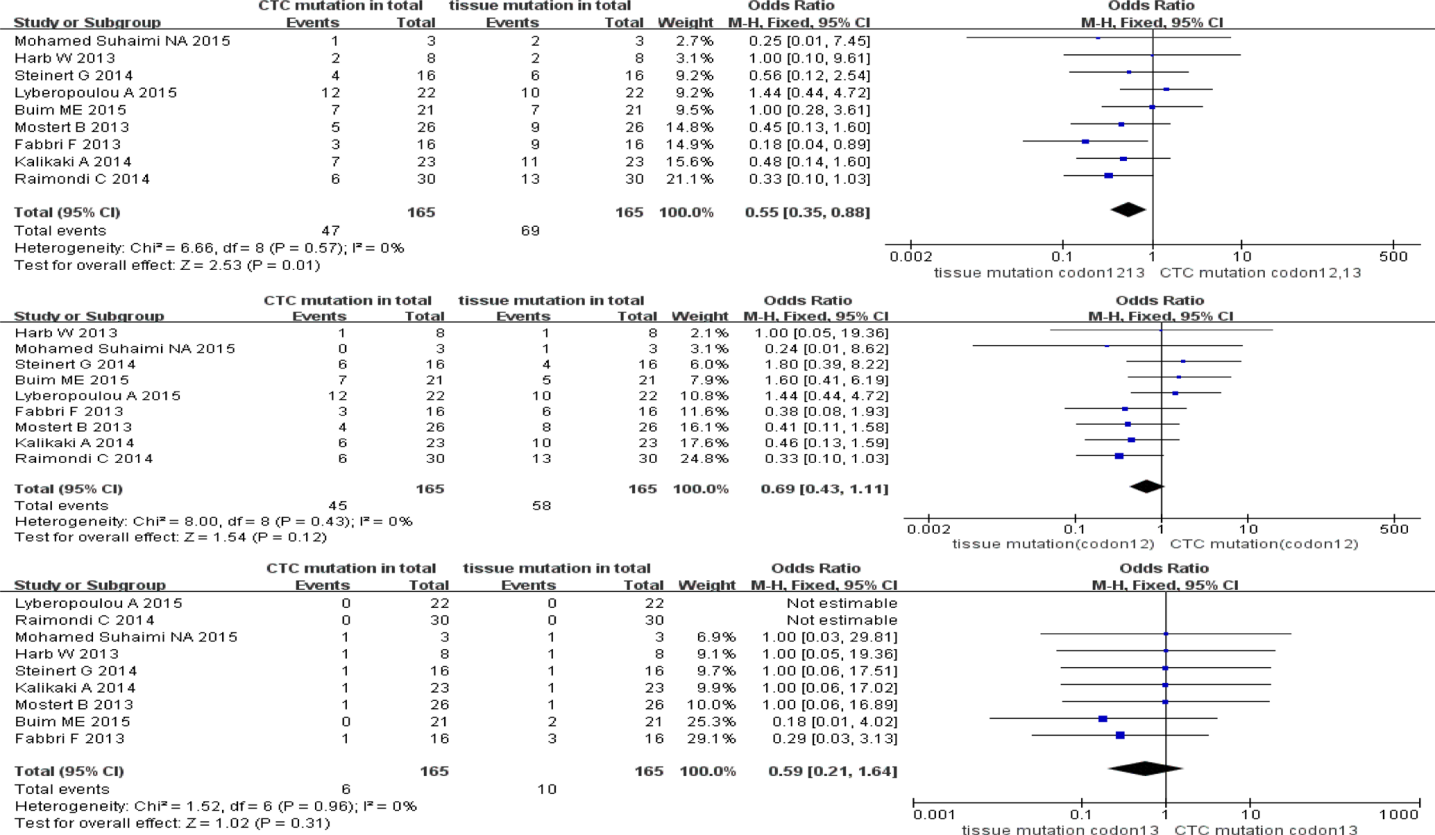
Pooled data analysis of KRAS codon12+13, codon12, codon13 mutation in paired CTCs and primary tumors (stage IV)

With reference to KRAS sub-type mutation in stage IV CRC patients, high homogeneity (all I^2^ = 0%) between studies and pooled ORs was observed with 0.55 (95% CI; 0.35, 0.88) for KRAS codon12+13 mutation, 0.69 (95% CI; 0.43, 1.11) for KRAS codon12 mutation, and 0.59 (95% CI; 0.21, 1.64) for KRAS codon13 mutation. Furthermore, only the KRAS codon12+13 mutation sub-type group presented discordance of mutation ratio in paired CTCs and primary tumors (*P* = 0.01 for codon12+13 mutation, *P* = 0.12 for codon12 mutation, and *P* = 0.31 for codon13 mutation) (Figure 3).

### Pooled data analysis of KRAS sub-type mutation in paired CTCs and primary tumors (stage III)

**Figure 4:**
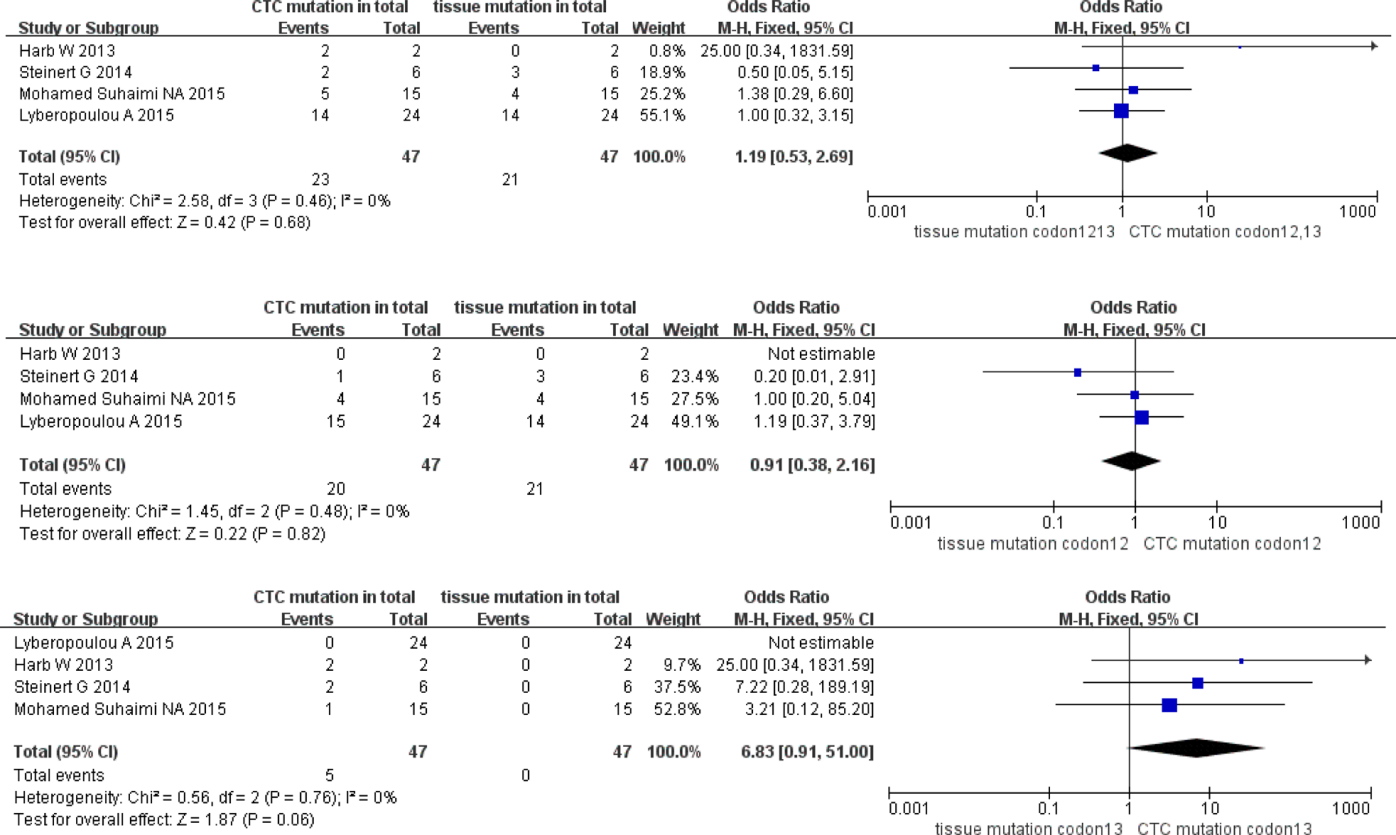
Pooled data analysis of KRAS codon12+13, codon12, codon13 mutation in paired CTCs and primary tumors (stage III)

The pooled ORs of KRAS sub-type mutation of paired CTCs and primary tumors in stage III were 1.19 (95% CI; 0.53, 2.69) for KRAS codon12+13 mutation, 0.91 (95% CI; 0.38, 2.16) for KRAS codon12 mutation, and 6.83 (95% CI; 0.91, 51.00) for KRAS codon13 mutation. All three KRAS sub-type groups showed high homogeneity (I^2^ = 0) of studies and concordance of mutation ratio in paired CTCs and primary tumor cohorts (test for overall effect *P* range from 0.06 to 0.82) (Figure 4).

### Pooled data analysis of KRAS sub-type mutation in paired CTCs and primary tumors (stage I + II)

**Figure 5:**
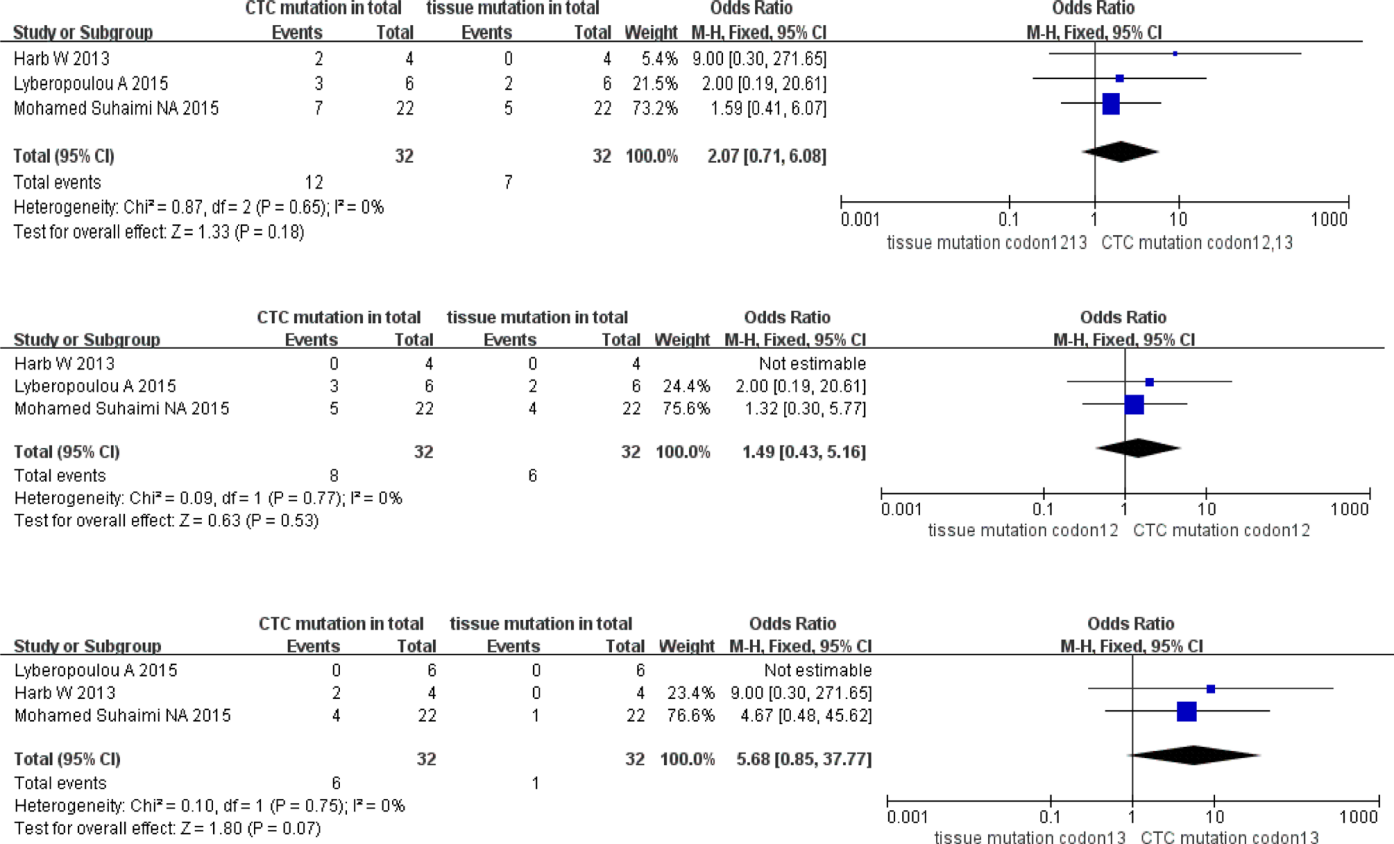
Pooled data analysis of KRAS codon12+13, codon12, codon13 mutation in paired CTCs and primary tumors (stage I–II)

The total ORs of KRAS sub-type mutation in paired CTCs and primary tumors at stage I+II were 2.07 (95% CI; 0.71, 6.08) for KRAS codon12+13 mutation, 1.49 (95% CI; 0.43, 5.16) for KRAS codon12 mutation, and 5.68 (95% CI; 0.85, 37.77) for KRAS codon13 mutation. However, no statistic divergence was observed in paired CTCs and primary tumors despite high homogeneity in all three KRAS sub-type groups (all I^2^ = 0% with *P* = 0.18 for codon12+13 mutation, *P* = 0.53 for codon12 mutation and *P* = 0.07 for codon13 mutation) (Figure 5).

### Pooled data analysis of subgroup stratified as enrichment methods of CTC, methods for detecting CTC mutations, and cutoff number of CTCs (stage IV)

We classified the qualifying studies based on enrichment approach of CTCs into a subset of six studies with Epcam-targeted and three without Epcam-targeted CTC enrichment. We stratified such subsets for further analysis. As a result, ORs in paired CTCs and primary tumors were 0.58 (95% CI: 0.34, 0.99) and 0.47 (95% CI: 0.19, 1.19) for the subsets with and without Epcam-targeted enrichment, respectively. We observed low heterogeneity and significant discordance in studies using the Epcam-targeted enrichment approach (I^2^ = 0%, *P* = 0.05) (Figure 6A). In parallel, we divided the methods for detecting mutations in CTCs into PCR-based (OR 0.57, 95% CI: 0.34, 0.96) and pyrosequencing-based (OR 0.45, 95% CI: 0.08, 2.43) subgroups. We found low heterogeneity in studies and divergence of KRAS mutation ratio in the PCR-based subgroup (I^2^ = 0%, *P* = 0.04) (Figure 6B). Finally, the cutoff number of CTCs was used to classify the studies as 1–2 CTCs (OR 0.64, 95% CI: 0.37, 1.09) and ≥ 3 CTCs (OR 0.38, 95% CI: 0.16, 0.92) subgroups. We found low heterogeneity in studies with significant difference of OR in the ≥ 3 CTCs subgroup (I^2^ = 0%, *P* = 0.03) (Figure 6C).

**Figure 6A:**
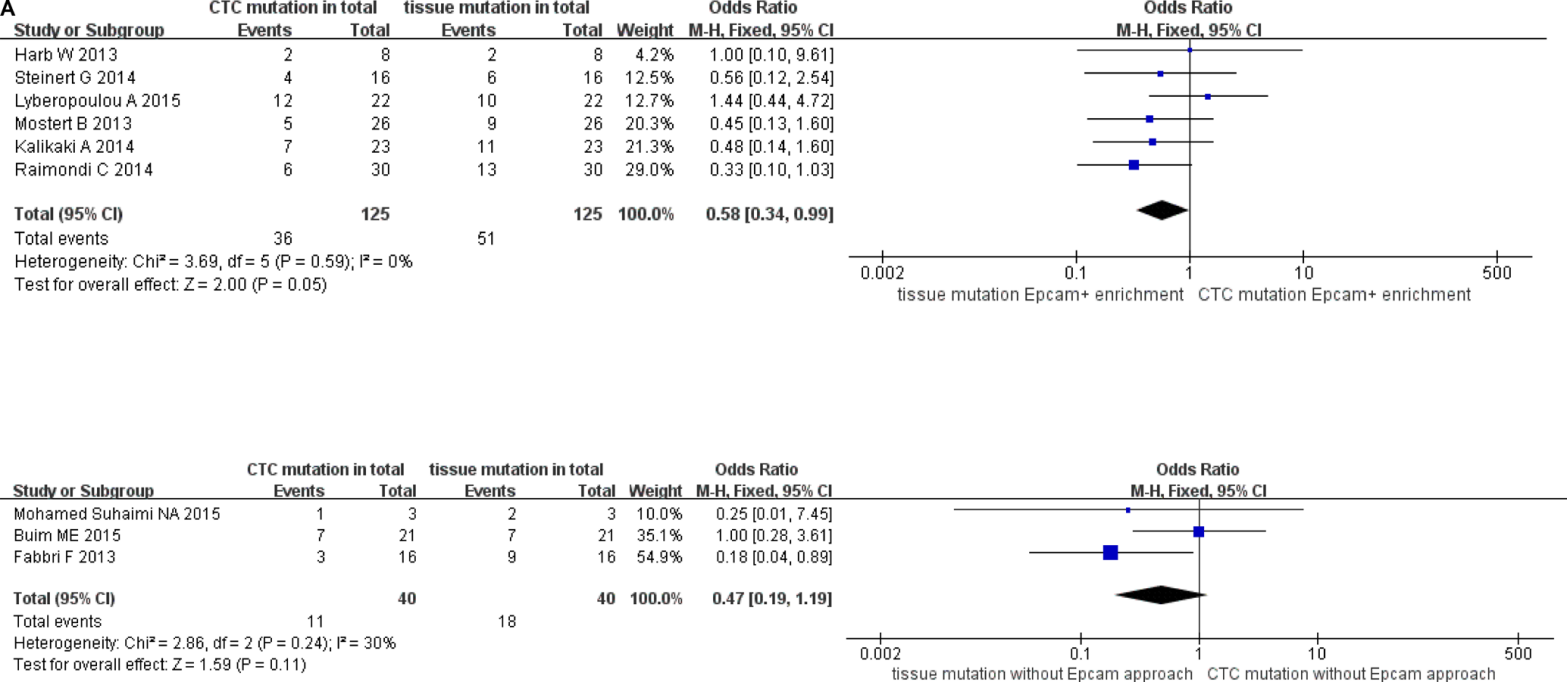
Subgroup analysis based on enrichment method of CTC in paired CTCs and primary tumors (stage IV)

**Figure 6B:**
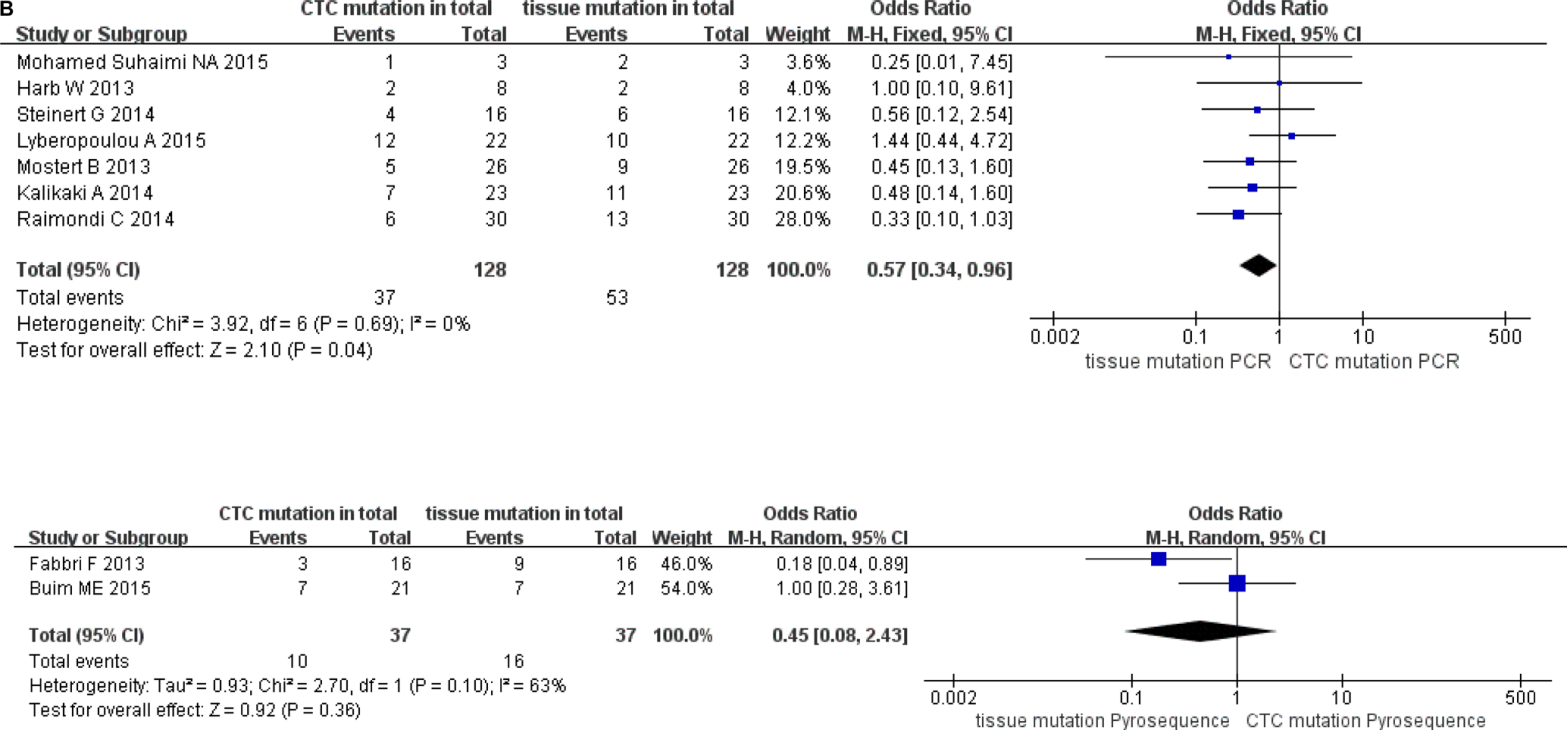
Subgroup analysis based on methods for detecting CTC mutations in paired CTCs and primary tumors (stage IV)

**Figure 6C:**
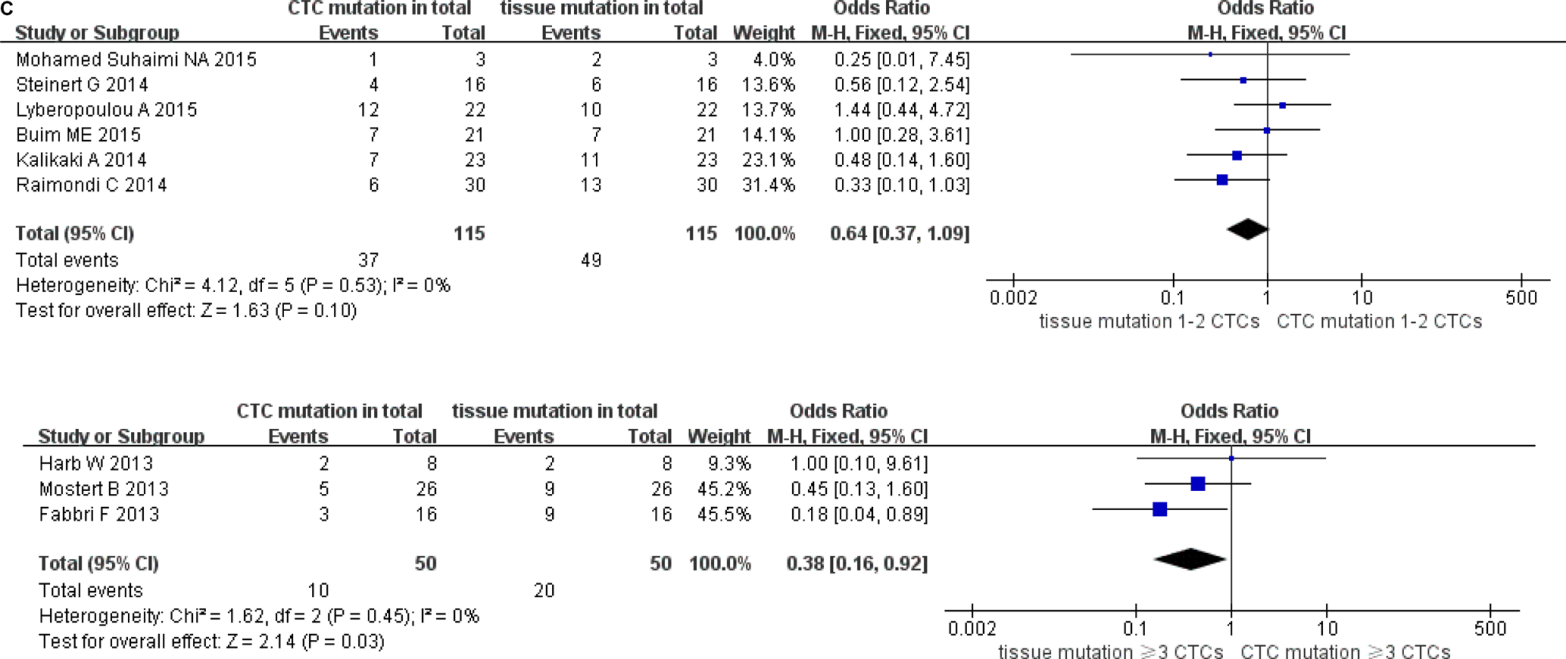
Subgroup analysis based on cutoff number of CTCs in paired CTCs and primary tumors (stage IV)

### Data analysis of BRAF mutation in paired CTCs with primary tumors

A total of 137 cases from four studies were analyzed based on detection of BRAF mutations in paired CTCs with primary tumors of CRC patients. The pooled OR was 0.44 (95% CI: 0.14, 1.11); however, we found no difference in paired subgroup populations with high homogeneity between studies (I^2^ = 0%, *P* = 0.08) (Figure 7A).

**Figure 7A:**
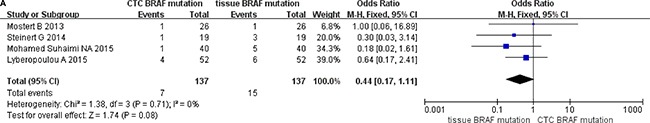
Data analysis of BRAF mutation in paired CTCs and primary tumors

### Data analysis of tumor status in CTCs with KRAS mutation

By comparing the differences between the tumor progression and the stable cohorts in CTCs with KRAS mutation, the risk ratio associated with different tumor status was 1.59 (95% CI; 0.69, 3.69). We saw no difference between studies, and measured low heterogeneity (I^2^ = 26%, *P* = 0.28) (Figure 7B).

**Figure 7B:**
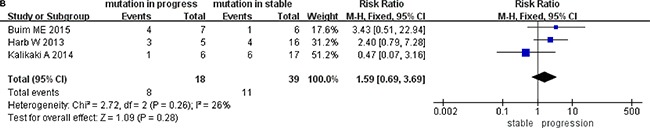
Data analysis of tumor status in CTCs with KRAS mutation

## DISCUSSION

Our meta-analysis compared the discordance of both KRAS and BFRF mutation in paired CTCs with primary tumors from CRC patients. Variations in genotype and phenotype between primary tumors and metastasis are effective prognosis biomarkers [15, 41, 42]. However, because of lacking specific tumor biomarkers and the limitations of molecular profiling technology for single cell analysis, CTC-related mutations in CRC are still under active investigation. Even though numerous studies correlated CTC counts with prognosis, we found here through our meta-analysis of the literature that few mutation-centric CRC studies focus on CTCs. From nine studies included, only one study compared KRAS and BRAF mutations in primary tumors, and CTCs and metastatic lesions. However, the data of mutations from metastatic lesions were not included in our meta-analysis because no other similar studies were available to carry out statistics [30]. Additional studies comparing the mutational status of CTCs combined with primary tumors and metastatic lesions are required in future CRC-related analysis.

Our findings here revealed discrepancies between KRAS mutations in CTCs and paired primary tumors by pooled meta-analysis. Even though high concordance between primary tumors and clinical metastases has been previously demonstrated, discordant sub-populations of genetic mutation intra-tumor or during the metastatic process are commonly reported [10, 14, 21, 43]. We grouped individual data drawn from each qualifying study into stage IV, stage III, and stage I + II subgroups. We observed high discordance of mutation in CTCs with paired primary tumors in the stage IV and III subgroups, demonstrating that CTCs do not always show genotype agreement with primary lesions, especially in metastatic patients [14, 18–20]. However, the limited number of samples from only three studies in the early stage subgroup, as well as few CTCs enriched in peripheral blood, decreased the confidence of our analysis for the stage I + II subgroup. Therefore, a larger cohort will be necessary to explore the molecular properties of CTCs and their relationship with primary tumors in CRC patients at early stages.

Recently, clinical and experimental findings demonstrated that in human CRCs, KRAS codon12 mutations were much more frequent in metastatic than in non-metastatic tumors, and were more aggressive than KRAS codon13 mutation [44–46]. Our findings revealed a higher frequency for codon12 mutations than for codon13 mutations in both CTCs (69 cases:14 cases) and primary tumors (84 cases:12 cases), in agreement with previous findings [16, 44–46]. Additionally, we found more KRAS mutations in CTCs than in primary tumors at early stage (stage I + II), the same in stage III, but fewer in stage IV. This higher frequency of KRAS mutations that we observed in CTCs may correlate with their aggressiveness and resistance to anti-tumor therapy. Our analysis demonstrated more KRAS mutations in CTCs than in tumor tissues at an early stage, but lower KRAS mutations in advanced stages of CRC. Furthermore, the decrease of KRAS codon12 mutations in CTCs at stage I–II vs stage IV suggested that codon12 mutations may be a more sensitive biomarker for dynamically tracking mutation variation in CTCs.

According to recent reports, Epcam- CTC can be more aggressive than Epcam+ CTC, but the EMT process renders Epcam- CTCs undetectable, which decreases the clinical application of molecular profiling for CTC enrichment based on Epcam [47–49]. To decrease selection bias, we performed subgroup analysis based on Epcam expression in stage IV patients. The six Epcam-targeted subgroups showed disagreement between CTCs and paired tumor tissues in stage IV patients, suggesting that target Epcam enrichment might be more suitable for identifying aggressive sources of CTCs with mutations than profiles based on size and density gradient. A similar result was found through gene sequencing and RT-PCR. PCR-based techniques for detecting genetic alterations have been proposed to provide advantages such as high sensitivity and specificity, albeit at a high cost [50–53]. Low-level mutations sometimes cannot be detected by conventional Sanger sequencing when they are masked by a high background level of wild-type [50]. The ≥ 3 CTCs subgroup showed significant discordance of CTCs with paired tumor tissues, suggesting that increased numbers of CTCs may account for increased mutations. Based on our analyses, Epcam-targeted CTC enrichment, PCR-based mutation profiling, and ≥ 3 CTCs enrichment may improve comparisons in mutation variation between CTCs and paired tumor tissues [36, 48–50, 53]. In addition to KRAS mutations, BRAF mutations are reported only in 8%–15% of CRC cells and are associated with poor response to anti-EGFR therapy [8, 54]. We also analyzed discordance of BRAF V600E mutation between CTCs and paired primary CRC tumors but detected no differences. Due to the low proportion of BRAF mutation in primary tumors and CTCs, BRAF mutations were not very significant to our meta-analysis. Considering the small number of studies included in our analysis, further studies with larger cohorts are warranted.

We observed a high concordance of KRAS status was observed in CTCs and primary tumors, contrary to discrepancies reported in previous studies [30, 36, 37]. We also analyzed clinical stage, mutation sub-type, methods of CTC enrichment, mutation profiling, and cutoff number of CTCs to decrease bias between studies. However, certain limitations must be considered when interpreting our results. First, only nine studies were included in our meta-analysis, with few samples per study further limiting subgroup analyses. Second, each study had insufficient samples regarding various characteristics such as stage, mutation sub-type, cutoff number of CTCs, CTC enrichment methods, and mutation profiling, which yielded few samples in each subgroup. However, homogeneity was observed in stage IV subgroups and other KRAS sub-types, suggesting the necessity of subgroup partition based on stage and codon type in future studies. Third, publication bias might affect our results even though no significant publication bias was observed in our meta-analysis. Despite these limitations, our meta-analysis evaluated the effects of KRAS and BRAF mutation in CTCs of CRC patients by using all qualifications with intensive subgroup analyses. Therefore, large sample studies of CRCs focusing on more detailed characteristics are urgently needed to investigate genotype profiling not only regarding mutational variations, but also tumor progression and prognosis. In conclusion, our analyses have provided insight into the molecular profiling and genetic detection of mutational variations in paired CTCs and primary tumors. We found mutation discordance between CTCs and primary CRCs, particularly in the stage IV and KRAS subgroups. Large sample studies stratified by clinical stage and KRAS sub-type are warranted to accurately evaluate mutational variations in CTCs compare to primary and metastatic CRC cells.
